# *Botryllus schlosseri* as a Unique Colonial Chordate Model for the Study and Modulation of Innate Immune Activity

**DOI:** 10.3390/md19080454

**Published:** 2021-08-09

**Authors:** Oron Goldstein, Edna Ayerim Mandujano-Tinoco, Tom Levy, Shani Talice, Tal Raveh, Orly Gershoni-Yahalom, Ayelet Voskoboynik, Benyamin Rosental

**Affiliations:** 1 Regenerative Medicine and Stem Cell Research Center, The Shraga Segal Department of Microbiology, Immunology, and Genetics, Faculty of Health Sciences, Ben Gurion University of the Negev, Beer Sheva 8410501, Israel; Orongold@post.bgu.ac.il (O.G.); eamanti24@gmail.com (E.A.M.-T.); shanital@post.bgu.ac.il (S.T.); orlyge@post.bgu.ac.il (O.G.-Y.); 2 Laboratory of Connective Tissue, Centro Nacional de Investigación y Atención de Quemados, Instituto Nacional de Rehabilitación “Luis Guillermo Ibarra Ibarra”, Calzada Mexico-Xochimilco No. 289, Col. Arenal de Guadalupe, Tlalpan, Mexico City 14389, Mexico; 3 Institute for Stem Cell Biology and Regenerative Medicine, Stanford University School of Medicine, Hopkins Marine Station, Stanford University, Chan Zuckerberg Biohub, Pacific Grove, CA 93950, USA; levyt@stanford.edu (T.L.); tal6933@stanford.edu (T.R.); ayeletv@stanford.edu (A.V.)

**Keywords:** immune tolerance, allorecognition, stem-cell transplantation, *Botryllus schlosseri*, tunicates, innate immunity, immune rejection, immune modulation

## Abstract

Understanding the mechanisms that sustain immunological nonreactivity is essential for maintaining tissue in syngeneic and allogeneic settings, such as transplantation and pregnancy tolerance. While most transplantation rejections occur due to the adaptive immune response, the proinflammatory response of innate immunity is necessary for the activation of adaptive immunity. *Botryllus schlosseri*, a colonial tunicate, which is the nearest invertebrate group to the vertebrates, is devoid of T- and B-cell-based adaptive immunity. It has unique characteristics that make it a valuable model system for studying innate immunity mechanisms: (i) a natural allogeneic transplantation phenomenon that results in either fusion or rejection; (ii) whole animal regeneration and noninflammatory resorption on a weekly basis; (iii) allogeneic resorption which is comparable to human chronic rejection. Recent studies in *B. schlosseri* have led to the recognition of a molecular and cellular framework underlying the innate immunity loss of tolerance to allogeneic tissues. Additionally, *B. schlosseri* was developed as a model for studying hematopoietic stem cell (HSC) transplantation, and it provides further insights into the similarities between the HSC niches of human and *B. schlosseri*. In this review, we discuss why studying the molecular and cellular pathways that direct successful innate immune tolerance in *B. schlosseri* can provide novel insights into and potential modulations of these immune processes in humans.

## 1. Introduction

The study of the immunological mechanisms that allow animals to regenerate and to recognize allogeneic tissues is an important trait in the field of transplantation. In vertebrates, T cells are the principal effector cells of the adaptive immune system for the nonself recognition. Interestingly, innate cells are activated by inflammatory signals released by damaged or stressed cells resulting from the allogeneic transplantation; this innate immunity is necessary for T-cell activation and the concomitant rejection response [1,2]. For example, following HSC transplantation, the hyperactivation of natural killer (NK) cells results in NK cell-mediated production of proinflammatory signals that induces and sustains the T-cell-mediated graft-versus-host disease (GVHD) response [3,4]. Innate immunity also plays an important role in the tolerance of the embryo tissues during pregnancy. In this case, innate responses are highly associated with pregnancy disorders such as recurrent pregnancy loss, preterm birth, and preeclampsia [5,6,7,8,9,10,11].

To study the cellular and molecular mechanisms guiding innate immune systems for tolerance or rejection, we review the idea of studying an emerging model of a colonial chordate, *Botryllus schlosseri*. *B. schlosseri* has been studied for decades and has very interesting natural phenomena which are based on innate immune responses, from natural transplantation or rejection to synchronized program cell removal and whole-body regeneration. This organism is a tunicate; it is part of the closest sister group of to vertebrates, and it lacks lymphocyte-based adaptive immunity [12,13,14]. This enables the study of the cellular and molecular mechanisms that direct tolerance rejection and regeneration in a system that lacks T- and B-cell-mediated immunity. In the recent decade, new tools and advancements have been added to the model of *B. schlosseri*, from the genome project [15] and gene sets of different immunological phenomena to isolation of immune cells and HSC transplantation [16,17]. *B. schlosseri’s* genome (~600 Mbp) was fully sequenced and annotated in 2013 [15], finding that many protein-encoding genes share significant homology with at least 75% of human genes [15]. In this review, we explain the different natural innate immunity-based phenomena in *B. schlosseri* and how they relate to mammalian processes. Additionally, we review the tools and gene sets available to study on the cellular and molecular levels those immunological phenomena in vivo and in vitro using this unique model organism.

### Botryllus schlosseri

*B. schlosseri* is an invasive marine colonial tunicate from the phylum Chordata, which can now be found all over the world [18,19]. Tunicates were named after the gelatinous “tunic” structure that covers their body and were proposed by Charles Darwin as a fundamental clue in the evolution of vertebrates [20]; indeed, molecular phylogenetics studies found them to be the vertebrates’ closest living invertebrate relatives [21,22].

**Figure 1:**
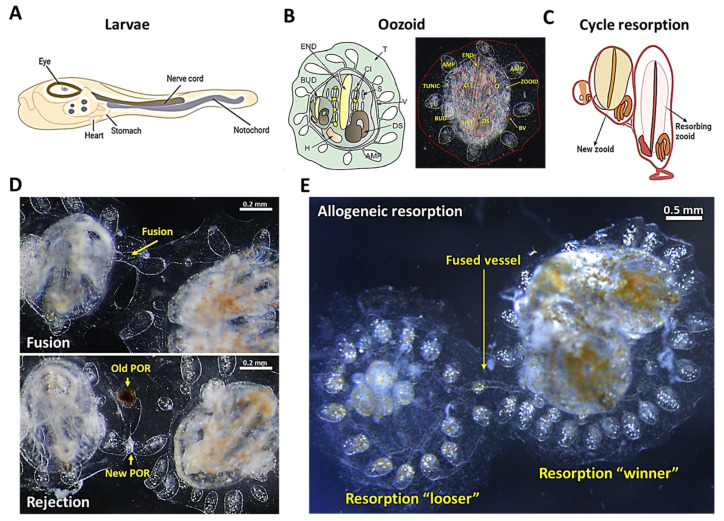
The anatomy of *B. schlosseri* and its different levels of naturally occurring immune responses. (**A**) Diagram of the tunicate larvae tadpole phase showing the nerve cord and the notochord. (**B**) Diagram and live imaging of the ventral view of a zooid (Z) and primary bud (BUD), embedded within a tunic (TUN) and connected with vasculature (V), which terminates in ampullae (AMP). The zooid has a branchial sac conformed by the endostyle (END), stigmata (S), cell islands (CI), digestive system (DS), and heart (H). (**C**) Diagram showing the “takeover” phase in the weekly cycle of zooid regeneration mediated by noninflammatory programmed cell removal of the resorbing old zooid. (**D**) Live imaging of two *B. schlosseri* colonies undergoing fusion (arrows show fused vasculature (top)) and rejection (arrows show points of rejection (POR) (bottom)). (**E**) Live imaging from the allogeneic resorption process, where one colony is the “loser” (which is resorbed), while the other is the “winner”, demonstrating normal developmental stages. (**A**,**C**) were created using BioRender; (**B**,**D**) were reproduced with permission from [17], Springer Nature Limited, Berlin, Germany, 2018; (**E**) was reproduced with permission from [16], National Academy of Sciences, 2016.

*B. schlosseri* has the ability to reproduce either sexually or asexually [23,24]. Following fertilization, a series of classic embryonic developmental stages over a 6 day period results in a tadpole larva conceived in the sexual pathway [23]. This larval stage is actually a major clue reflecting the close relationship to vertebrates, as it features characteristics such as a tail, notochord, neural tube, and striated musculature (Figure 1A) [15,22,23,25]. The swimming larva settles on a substrate within a few hours after hatching and metamorphoses into a sessile oozoid with an invertebrate-like body plan (Figure 1B), which initiates a cyclical blastogenic process that results in the formation of a colony of genetically identical zooids and buds [23,26]. The budding cycle takes 7 days and begins with secondary buds turning into primary buds which, at the end of the cycle, replace the previous generation of zooids, whose cells die through programmed cell death and are cleared through programed cell removal (Figure 1C) [17,23,27,28,29]. This cyclical budding process is mediated by stem cells, which sustain the organism throughout its life, and which are responsible for organogenesis in the asexual reproduction cycle and gametogenesis in the sexual reproduction pathway [17,30,31,32,33,34,35].

*B. schlosseri* has different levels of naturally occurring immune responses. When two genetically compatible colonies that share at least one allele in their *Botryllus histocompatibility factor* (*BHF*) gene touch, they fuse, share circulation, and form a chimera (Figure 1D, top) [36,37,38]. However, incompatible colonies, which do not share any allele in the *BHF* gene, undergo a rejection response, creating points of rejection between the colonies with necrotic tissue (Figure 1D, bottom) [31,33,34,36,37,38]. Interestingly, in fused animals on a semi-compatible level, in some cases, one chimeric partner is eliminated by an inflammatory process of allogeneic resorption which is comparable to human chronic rejection (Figure 1E) [16,39].

Another fascinating phenomenon that takes place when two compatible colonies fuse circulation is stem-cell competition, whereby stem cells from one colony infiltrate the reproductive organs of the other colony, overtaking gametogenesis and giving rise to progeny of its own genotype; thus, the stem cells represent biological units of natural selection. Stem-cell competition is an example of a new field that evolved from studying *B. schlosseri* chimeras. Cell competition in *B. schlosseri* was demonstrated in an experiment that used genetic markers to track the genotype of somatic and germline tissues within a two-colony chimera, revealing the expression of only one genotype in the germline tissues of both colonies, as well as one to few genotypes in somatic cells [31,33,34]. Follow-up studies further demonstrated that this clone takeover is mediated by stem cells [31]. In order to translate the study of stem-cell competition to mice, tetra-chimera mice were developed by injecting three distinct colored embryonic stem cells into blastocytes, each labeled with a different fluorescent reporter [40]. As the embryos developed, many adjacent seminiferous tubules yielded several fluorescent signals; however, only two to three different colors in each testicle remained after maturation [41]. The interpretation suggests that, while all clones entered the genital ridges bilaterally, only one of them remained in the adult mice [42]. In the blood-forming system, aging and disease processes have been attributed to stem-cell competition. During aging, there is an increase in the proportion of stem cells producing myeloid vs. lymphoid blood populations, which may be attributed to stem-cell competition, as observed in old versus young mice and humans [43,44,45,46,47]. Clonal expansion and stem-cell competition were clearly shown in the stepwise progression of aberrant preleukemic clones toward leukemias [48,49,50,51,52,53], as well as in the clonal expansion of smooth muscle cells in atherosclerosis in mice and humans [54,55]. These are examples of how studies in *B. schlosseri* have led to discoveries in mammalian development, aging, cancer, and atherosclerosis [42].

## 2. Natural Transplantation Phenomena (Fusion/Rejection Mechanisms)

**Figure 2:**
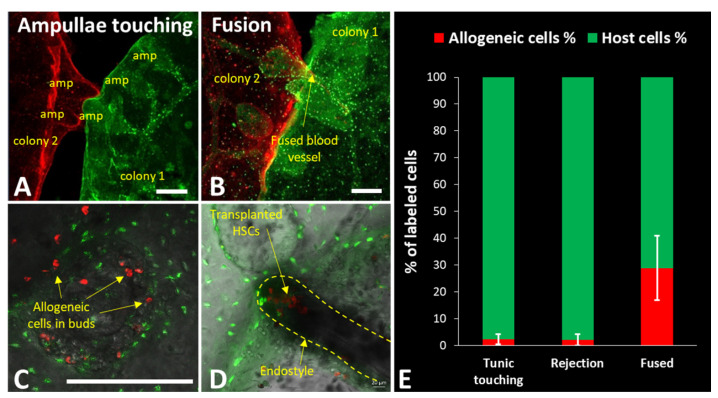
Transplantation in vivo using exogenous labeling in *B. schlosseri.* (**A**) Two colonies differentially labeled with CFSE (green) and CellTracker Deep Red, prior to fusion. (**B**) Fused colonies through the blood vessel and exchanged allogeneic cells can be seen in the confocal image of the live colonies. The transparent body of *B. schlosseri* is used to follow transplantation in vivo. (**C**) Transplanted allogeneic cells in a developing secondary bud, followed in a live colony. (**D**) Transplanted HSCs (exogenous lipophilic dye DiD, red) in the endostyle stem-cell niche in live animals. Bars = 200 µm. (**E**) Measurement by flow cytometry of transplanted allogeneic cell abundance 3 weeks after transplantation; average and SD of three pairs of touching animals without response, rejecting animals, and fused colonies. This shows the ability of measurement of transplantation success or modulation. (**A**,**B**,**D**) were reproduced with permission from [17], Springer Nature Limited, 2018; (**C**) was reproduced with permission from [16], National Academy of Sciences, 2016.

When two colonies of *B. schlosseri* touch with the ampullae (end point of their blood vessels), they experience a process of self/nonself recognition [36,38,56]. Colonies recognize each other as self and fuse their vasculatures to form a natural parabiont (Figure 1D top) if they share at least a single allele of the polymorphic histocompatibility gene, Botryllus histocompatibility factor (*BHF*) [38]. Upon the establishment of a common vasculature system, the immune cells and the stem cells can freely flow from one partner of the chimera to the other, resembling parabionts in mammalian experimental systems (Figure 2A–C). On the somatic level, chimerism can be stable for a long period of time, when both genotypes are present and visible. Since stem cells are also free to move within the chimera, a hierarchical competition of stem cells occurs in the chimeric colony where “winner” cells will be heritable dominant, giving rise to gonads of a single germline origin while maintaining somatic chimeras [33,34]. Heritable germline winner and loser strains reflect genetically determined stem-cell phenotypes [31,32,35]. Interestingly, in some cases of fused colonies, one of the partners will be resorbed. This usually happens during the blastogenic cycle, while there is a programmed cell removal of the old zooids; however, an inflammatory process also prevents the developmental process of the new zooids from buds [16,39]. This process is termed allogeneic resorption (Figure 1E).

Genetically noncompatible colonies undergo an immune rejection response where inflammatory and cytotoxic cells are involved, creating necrotic areas between the touching ampullae (Figure 1D bottom) [17,36,38]. The basis for the cytotoxicity rejection response is represented by cytotoxic morula cells (MCs), which seem to work according to the “missing self-hypothesis”, resembling human natural killer (NK) cells. Without the inhibitory recognition of compatible *BHF*, MCs kill the target cells, resulting in a necrotic lesion at the points of rejection (Figure 1D bottom) [17]. This allorecognition is ascribed as the invertebrate counterpart of transplantation immunity [16].

## 3. Zooid Resorption and Regeneration as a Model for Programed Cell Removal

A *B. schlosseri* colony evolves from a single tadpole-like larva that developed through classical chordate embryogenesis from a fertilized egg. Blastogenesis begins when the larva metamorphoses into a filtering oozoid that carries a bud, which develops into an individual adult zooid. Zooids grow buds that develop and replace them every week, forming colonies (Figure 1A–C). Each colony is composed of filtering adults (zooids), primary buds, and secondary buds. The blastogenetic cycle is defined by changes across these three generations which occur every week at 20 °C [24].

Zooids live for 1 week and get resorbed when the new generation replaces them. This “takeover” event is mediated by the coordination of different molecular and cellular processes, in which the circulating blood cells are involved. Before the takeover takes place, a diffuse programmed cell death is triggered, from the anterior to the posterior of the zooid [57], by a mitochondria-dependent apoptotic pathway. This has been evidenced by an increase in chromatin condensation [27], the activation of caspases (caspase-3 and -9) [58], the overexpression of mammalian apoptotic molecules (i.e., Bax, Fas, and FasL), and the downregulation of the antiapoptotic Bcl-2 [59]. Phosphatidylserine and oxidized lipids are expressed on the plasma membrane surface of affected cells as phagocyte recognition signals [27]. In the next steps of the takeover, circulating phagocytic cells (positive to CD36 antibodies) infiltrate senescent tissues, engulf apoptotic cells, and then also die by apoptosis (Figure 1C) [27,60].

Zooid resorption generates a substantial amount of biological material, which is recycled by developing buds to maintain blastogenic development [61]. The resorption of zooids and the allogeneic resorption (detailed in part E) that takes place in *B. schlosseri* chimeras share similarities in the immune response, as both involve the constant crosstalk among apoptosis, self/nonself recognition, and phagocytosis [62]. As this crosstalk occurs under natural conditions, *B. schlosseri* represents a valuable model to study, at different levels (genes, metabolites, signaling molecules, and cellular functions), the mechanisms that guide programmed cell removal, which enables the regeneration of new zooids.

## 4. *Botryllus schlosseri* as a Model to Study the Innate Immunity

*B. schlosseri* has an efficient immune system that not only fights and prevents infections, but also orchestrates histocompatibility, incompatibility, rejection, and zooid resorption processes. Interestingly, when its genome was annotated and compared with invertebrate and vertebrate genomes, it was found that numerous genes associated with immune system and hematopoiesis, including *ZBTB1*, *MEFV*, *DSG3*, *NQ01*, *NQO2*, and *BHLHE40*, which are involved in leukocyte development, as well as an additional set of genes that could attributed to precursors of human hematopoietic lineages, could be detected in *B. schlosseri* but not in other invertebrate species or solitary tunicate species [15].

Immune responses in *B. schlosseri* are mediated by homeostatic cell turnover and the licensing of innate cytotoxic cells, which collaborate with activated phagocytes. These immune effector cells are circulating blood cells that currently exhibit phagocytic and cytotoxic cell activity. Among phagocytic cells, *B. schlosseri* has a myeloid lineage that shares a large set of genes with mammalian myeloid lineages [17]; it also has amoebocytes and large phagocytes which are morphologically more related to hemocytes of arthropods and echinoderms [63]. These phagocytes engulf microorganisms and damaged self-cells, contain phagosomes with hydrolytic enzymes, lipids, and lipofuscins [64], and can be subdivided into static (in the circulatory system epithelia) and mobile (circulating through the colony) populations [65].

MCs of *B. schlosseri* have been characterized as cytotoxic cells; they are the most abundant circulatory cell type and have large cytoplasmic granules containing an inactive form of phenoloxidase (PO) [64]. Gene expression analysis has shown that they express a tunicate-specific gene repertoire and a set of genes (15%) sharing homology with vertebrate lymphocytes [17,63].

At the molecular level, BsTLR1 is expressed in both phagocytes and MCs of *B. schlosseri* as a member of the TLR receptor family, which is actively involved in self/nonself recognition [66]. Blood circulating cells also express a gene of a type II transmembrane protein that is related to CD94 and NKR-p1 receptors of human NK cells and T lymphocytes. This protein has been found to be upregulated during the allorecognition process [67]. Rhamnose-binding lectin (BsRBL) has been identified as an effector molecule that activates phagocytes, thus inducing the release of cytokine-like molecules that are recognized by the anti-IL1α and anti-TNFɣ antibodies [68]. Furthermore, activated phagocytes signal through Ras-like small GTPases, MAPKs, and NF-κB networks to trigger the recognition response of foreign cells [69].

The work in *B. schlosseri* has guided us to hypothesize that innate immune mechanisms during tissue maintenance, allorecognition, and regeneration are conserved and highly important for the initiation of the adaptive immune response in mammals. Therefore, this model allows the study of the orchestrating cellular and molecular processes around these immune responses, focusing on the innate immune responses. This information can then be translated to human immunity, with a particular impact on the improvement of therapeutic strategies for stem cells, tissue, and organ transplantation.

Moreover, the immune defenses of tunicates have made them a potential source of various natural drug resources with great potential for pharmacological applications. For example, the hemagglutinating activity of lectins in ascidian hemolymph has an important immune role [70]; marine lectins have been investigated as potential antimicrobial and antiviral agents, as well as compounds with immunomodulatory and cytotoxic effects on tumor cells [71]. Five homologous transcripts of rhamnose-binging lectins (RBLs) have been identified to enhance phagocytosis in *B. schlosseri* [70]. Furthermore, components of the complement alternative pathway (C3 and Bf orthologs) [72,73] and components of the lectin pathway (mannose-binding lectin, ficolin, and mannose-associated serine protease 1) are transcribed by MCs and associated with nonself recognition, opsonization, and clearance of microbes and apoptotic cells [74].

The previously mentioned PO enzyme, which is degranulated and released by *B. schlosseri* MCs, is a bioactive molecule with cytotoxic activity against microbial infections (i.e., yeast cells and bacterial spores), as well as nonself cells (i.e., incompatible blood) [70,75]. MCs are also the main source of the soluble cytokine-like proinflammatory molecules IL-1-α and TNF-α, which are suggested to be released in the presence of incompatible cells and microbes [70]. Further studies are needed to explore the drug potential of these *B. schlosseri* molecules and their possible pharmacological applications.

Several other compounds with antifungal, antidiabetic, antioxidant, and antitumor potential have been identified in tunicates. More in-depth information about this topic was reviewed in [76,77].

## 5. *Botryllus* as a Model for HSC Transplantation

*B. schlosseri’s* attributes have shown many similarities to vertebrates and mammals, whether in its blood circulation, stem-cell biology, or immune characteristics [31,33,56]. Similarly, to vertebrates, *B. schlosseri* stem cells reside in unique niches, where their status is assumed to be regulated on a spectrum where one end represents quiescence and the other represents differentiation/expansion. In 2005, Laird et al. showed stem-cell-based transplantation [31]; in 2008, Voskoboynik et al. successfully identified the endostyle niche and isolated somatic stem cells of *B. schlosseri* from it (Figure 2D) [35]. This advancement led to further studies focused on characterizing the niche and the stem cells.

HSCs, which are at the top of the hierarchy when it comes to blood/immune cells, maintain the organism’s blood and immune systems throughout its lifetime. They have been thoroughly studied in several organisms, the most important of which are humans and mice [78,79,80,81]. In 2018, Rosental et al. [17] successfully mapped whole transcriptomes of cells and tissues in the hematopoietic system of *B. schlosseri*, such as HSCs and their niche (endostyle), progenitor cells, and immune effector cells. They sorted 23 separate populations, mapped their transcriptomes, and identified a cluster of cells in which 235 genes were differentially upregulated, showing a significant gene activity homology to human and mammalian HSCs. Through transplantation assays, they showed those that enriched HSC differentiation to other *Botryllus* blood cells, as well as those home to the endostyle-niche (Figure 2D). For the characterization of the endostyle as an HSC niche, they compared transcriptome data from 10 endostyles to 34 whole colonies, finding 327 genes that were significantly elevated and shared with the upregulated genes in mouse and human hematopoietic bone marrow. This suggests a common origin for the endostyle niche in *B. schlosseri*, as well as the vertebrate hematopoietic bone marrow, beyond the homology HSCs and myeloid lineage-derived immune cells [17,63].

This recent work, taken together with previous research, makes *B. schlosseri* a complete model for HSC transplantation, considering their ability to isolate the HSCs, their interaction with the immune effector cells, and their localization to the HSC niche (Figure 2D). This includes the ability to analyze the level of transplantation-induced chimerism by flow cytometry (Figure 2E).

## 6. Allogeneic Resorption as a Model for Chronic Rejection

In many cases, after the fusion between two colonies of *B. schlosseri*, one of the semi-compatible partners will get reabsorbed within several weeks through an inflammatory process that prevents the regeneration of the new zooids (Figure 1E) [39]. The driving mechanisms of this process were elucidated by Corey et al., who identified cytotoxic MC as a key immune effector cell type in the process of allogeneic resorption [16]. The presence of MC resulted in gene expression changes in the “losing” partner that trigger cell-death programs and developmental processes defects. When allogeneic MCs were adoptively transplanted to colonies, they caused an inflammatory response preventing the development of next-generation zooids (in comparison to mock injections or non-MC donors), demonstrating that MCs are the drivers of this process. The isolation and RNA-sequencing of buds and zooids from resorbing versus non-resorbing parts of the chimeras provided a list of differentially expressed genes, revealing an upregulation in the expression of regulatory genes of host defense and proinflammatory markers.

Genes related to the complement system, such as *MASP1*, *MASP2*, and C3, were identified to have expression changes during allogeneic resorption, along with TNF-associated proteins (*TRAF3* and *TRAF4*), coagulation components (*KLKB1*, *KLK3*, *F2*, *F8*), cell death (CASP2/7/9), and lysosomal proteinases (*CTSV*, *CTSF*). These data confirm that the process of allogeneic resorption comprises different crosstalk events which can be further studied in *B. schlosseri*.

The interleukin family member IL-17 resulted to be a key upregulated gene (60-fold increase). IL-17 is secreted by innate immune cells and is involved in the clearance of fungi and extracellular bacteria [82]. IL-17 acts as a key regulatory cytokine, and its upregulation results in tissue damage due to excessive inflammation, chronic inflammation [83], autoimmunity [84], and chronic GVHD in higher vertebrates [85]. In comparison, in *B. schlosseri*, the use of recombinant *B. schlosseri* IL-17 protein led to a significantly upregulated cellular cytotoxicity in a dose-dependent manner [16]. This result, taken together with the gene sets showing the upregulation of classical inflammatory responses, shows the parallels to human chronic rejection.

## 7. Prospect of a General Allogeneic Model

Currently, allogeneic HSC transplantation is used to treat several diseases in humans; however, this clinical process is highly complex and requires prophylactic treatment to prevent immune rejection [86]. Some of the more prominent options for prophylaxis include calcineurin inhibitors, rapamycin, mycophenolate mofetil with (or without) anti-thymocyte globulin, or, in the case of acute GVHD, use of systemic corticosteroids like methylprednisolone [87]. Despite recent advancements and the use of prophylaxis, acute GVHD is diagnosed in close to half of the allogeneic HSC transplantation procedures, and it is associated with poor prognosis [88]. Chronic rejection is only exacerbated by resistance to corticosteroid treatment; the inability to control chronic rejection leads some patients to require a re-transplantation which increases clinical risks [88]. In higher vertebrates, T cells have a major role in chronic rejection, GVHD, and pregnancy disorders [89,90]. Elucidating the immune-related mechanisms behind the activation of these effector cells in an allogeneic setting will give us a better understanding of how to circumvent their cytolytic activation and positively modulate the process of chronic rejection. NK cells and T cells in humans share the characteristic of identifying allogeneic self/nonself and are activated by either the identification of nonself or the lack of self.

In *B. schlosseri* allogeneic rejection occurs in a similar fashion to vertebrates, despite its more innate immune-based system. As previously discussed, BHF in *B. schlosseri* shares some attributes with human MHC [91], in that its recognition as “self” leads to a major inhibitory mechanism of cytotoxicity in allorecognition. BHF’s inhibitory effects on cytotoxicity [17], combined with observational evidence of fusion of colonies that share at least one BHF allele, suggest that the mechanism of cellular toxicity during allorecognition in this tunicate stems from the ‘missing self’ and can be compared to recognition by NK cells in advanced vertebrates [63]. Taken together, these findings demonstrate similarities in the innate immune responses between *B. schlosseri* and humans on the cellular and molecular levels. While the driving force in each organism is different, points of interaction such as immune pathways and key recognition molecules could be studied in an easy access model and then translated into mammalian complex models.

## 8. Conclusions

The advancements in research and scientific tools in the *B. schlosseri* model have promoted *B. schlosseri* as an interesting model to study innate immune system responses, more specifically in transplantations. One of the more prominent research tools, the *Botryllus* genome project [15], enabled analysis on the genetic and molecular levels, thus identifying the *BHF* [38] on the fusion histocompatibility locus [92]. Moreover, the genome project confirmed the location of the tunicates as the closest invertebrate group to vertebrates, whereby many immune genes are shared with mammals and their hematopoietic system [15].

Furthermore, as mentioned above, *B. schlosseri* has natural occurring phenomena which resemble many basic immunological processes, such as (I) rejection as acute rejection in transplantation, (II) fusion as natural parabionts that share stem cells, (III) stem-cell competition and chimerism, (IV) natural weekly cycle of zooid resorption and new bud development, working through classical programmed cell removal and regeneration mechanisms comparable to vertebrates, and (V) allogeneic resorption as a chronic rejection process.

Moreover, there were several gene expression sets obtained during the research of the above-described processes, which enabled the search of candidate genes and pathways that affect those immune-associated processes, for example, gene sets of allogeneic resorption [16], fusion and histocompatibility-associated genes [38], 23 different cellular populations and endostyle [17], and zooid regeneration and developmental processes [23].

Taken together, the tools and advancements in *B. schlosseri*, along with the ability of in vivo and ex vivo cellular immune profiles, with cellular and molecular manipulations (from morpholinos to recombinant proteins), represent the foundation for future discoveries on immune activation mechanisms in a simple model, which is relevant for human immune research.
